# Promesas periféricas: expectativas, coaliciones y vacunas covid-19 en Argentina

**DOI:** 10.1590/S0104-59702025000100008

**Published:** 2025-04-07

**Authors:** María Cecilia Sanmartin, Gabriela Bortz

**Affiliations:** i Becaria doctoral, Escuela de Economía y Negocios/Universidad Nacional de San Martín. San Martín – Buenos Aires – Argentina. orcid.org/0000-0001-5967-7284 mceciliasanmartin@outlook.com; ii Investigadora asistente, Escuela de Economía y Negocios/Universidad Nacional de San Martín. San Martín – Buenos Aires – Argentina. orcid.org/0000-0001-7151-6686 gbortz@unsam.edu.ar

**Keywords:** Promesas tecnocientíficas, Expectativas, Vacunas covid-19, Coaliciones, Coproducción, Technoscientific promises, Expectations, Covid-19 vaccines, Coalitions, Coproduction

## Abstract

Las vacunas covid-19 se volvieron la gran promesa para enfrentar la crisis sanitaria, incluyendo en la periferia global. Este trabajo analiza la construcción y movilización de promesas tecnocientíficas para garantizar el acceso a las vacunas en Argentina. Basado en el análisis de notas periodísticas y entrevistas, en un abordaje en coproducción con los estudios sociales de la ciencia y la tecnología, se analizan coaliciones de actores clave, promesas tecnocientíficas y elementos cognitivos, simbólicos y materiales coordinados alrededor de “vacunas covid-19” que sustentan materialmente las promesas. Este trabajo permite profundizar en los repertorios de legitimación para la toma de decisiones, y busca mostrar cómo una promesa “periférica” puede cumplirse y materializarse.

## Abstract (en)

Covid-19 vaccines have shown great promise in addressing the health crisis, including in the global periphery. This work analyzes the construction and mobilization of technoscientific promises to ensure vaccine access in Argentina. Based on the analysis of newspaper notes and interviews, in an approach bringing together social science and technology studies, we examine coalitions of key actors, technoscientific promises, and cognitive, symbolic, and material elements coordinated around “covid-19 vaccines” that materially support these promises. This work allows us to delve deeper into the legitimization repertoires for decision-making and seeks to show how a “peripheral” promise can be fulfilled and materialized.

La pandemia de covid-19 puso a prueba la capacidad de los Estados de salvaguardar la salud de sus ciudadanos. Este desafío fue aún más crítico en los países en desarrollo, donde los recursos son limitados y las desigualdades en el acceso a la atención médica son evidentes (Faria, Alvarez, Santos, 2023; India’s…, 2021). Desde el inicio de la pandemia, las vacunas se convirtieron en la gran promesa para hacer frente a la crisis sanitaria global atrayendo la atención central de los esfuerzos de investigación y desarrollo (I+D). A nivel mundial, las principales compañías farmacéuticas generaron expectativas en torno al desarrollo de una vacuna contra la covid-19, compitiendo aceleradamente por captar el mercado (Balawejder, Sampson, Stratton, 2021; Zhang, 2021). Los desarrollos mostraron la concentración del diseño y producción de estas vacunas en países desarrollados, exacerbando las disparidades geopolíticas y de acceso a vacunas entre “centros” y “periferias” (Blinder, Zubeldía, Surtayeva, 2021; Sanmartin, 2023).

Argentina posee una más que centenaria tradición de investigación biomédica (Zabala, 2009) y más de cuatro décadas de trayectoria en biotecnología de excelencia (Kreimer, Zabala, 2006; Kreimer, 2010). Estas capacidades científicas contribuyeron a la consolidación del entramado nacional de empresas biotecnológicas (Stubrin, 2022). Estas se especializaron no solo en la copia de productos biotecnológicos intensivos en conocimiento (biosimilares), sino en innovación que permitió incrementar la competitividad del sector (Gutman, Lavarello, 2014; Stubrin, 2019). Las capacidades nacionales para la elaboración de productos biológicos para la profilaxis de enfermedades infectocontagiosas se remontan al Instituto Bacteriológico en 1912^1^ (Carbonetti, 2021; Zabala, Rojas, 2022). Las transformaciones en el mercado global de producción de vacunas, las regulaciones sanitarias y de propiedad intelectual, la destrucción de capacidades en laboratorios públicos, así como el histórico menor interés relativo de la industria biofarmacéutica en el mercado de las vacunas (Kominers, Tabarrok, 2022), han derivado en que la provisión de vacunas en Argentina muestre un sesgo importador, concentrado en empresas multinacionales (Corvalán, 2017).^2^ En términos de acceso a las vacunas, la responsabilidad del Estado nacional sobre el financiamiento y provisión puede rastrearse hasta la ley 1.420 de 1884, que estableció la obligatoriedad de vacunación en establecimientos escolares nacionales, y la ley 4.202 sobre vacunación y revacunación obligatoria de alcance nacional (Di Liscia, 2011).

Con el inicio de la pandemia, desde reactivos de diagnóstico, mascarillas y, especialmente, vacunas, estas tecnologías sanitarias reafirmaron su *status* político: tanto como tecnologías políticas, embebiendo visiones de Nación (Winner, 1987), así como emblema representativo de las políticas de los estados (Joerges, 1999) frente a la covid-19. La pandemia también marcó un replanteamiento del papel del Estado y la ciencia, así como de sus formas de intervención en la sociedad, reafirmando el compromiso histórico de la ciencia y la tecnología con la sociedad con la promesa de abordar problemáticas locales (Kreimer, Zabala, 2006; Kreimer, 2023).

En Argentina, la trayectoria de capacidades en investigación clínica (Romero, 2016) permitieron posicionar rápidamente al país como centro de pruebas para las vacunas desarrolladas por grandes empresas farmacéuticas. Las capacidades científicas e industriales permitieron al país tanto la firma de acuerdos para la producción local de componentes de estas vacunas, como posicionarlo como el primer país latinoamericano en desarrollar integralmente una vacuna contra la covid-19 aprobada por un organismo regulador de referencia internacional. Estas acciones implicaron una articulación flexible entre diversas coaliciones que involucraron al Estado, al sector científico y tecnológico público, al sector sanitario y al sector biofarmacéutico privado, movilizando elementos cognitivos, simbólicos y materiales en un contexto de expectativas en cuanto al acceso a tecnologías “útiles” (Bortz, Gázquez, 2020; Gazquez, Bortz, Santos, 2022).

¿Qué expectativas se movilizaron alrededor de las vacunas contra la covid-19? ¿Cómo se construyeron y coordinaron las coaliciones que materializaron estas expectativas en tecnologías vacunales “exitosas” en un país “periférico”? ¿Qué elementos sustentaron y legitimaron dichas coaliciones? Este trabajo analiza la construcción y movilización de promesas tecnocientíficas en torno a las vacunas covid-19 en Argentina. Específicamente, se reconstruyen las expectativas y estrategias desplegadas para garantizar el acceso a las vacunas en el país, y transformar el desarrollo íntegro de una vacuna en un objetivo legítimo y realizable en un país en desarrollo. Se examina cómo diversos actores clave participaron en la formación y circulación de estas expectativas y qué capacidades locales influyeron en el desarrollo de las distintas tecnologías vacunales para dar credibilidad a estas aspiraciones. Se reconstruyen también las coaliciones de actores y elementos materiales alineados para sustentar la gran promesa de acceder a la vacunación a tiempo y que permitieron construir el funcionamiento de los distintos artefactos “vacunas covid-19” que se sucedieron entre 2020 y la actualidad.

El trabajo utiliza una metodología de análisis documental basada en 150 artículos periodísticos publicados entre marzo del 2020 y agosto del 2023 y en 31 entrevistas realizadas a actores clave. El análisis se basó en los repertorios discursivos esgrimidos desde actores del gobierno nacional, de los sectores sanitario y científico-tecnológico y desde la industria biofarmacéutica local a partir de dichos artículos y entrevistas. A nivel teórico, el artículo parte de una visión de constitución mutua del orden social y material, propia de los estudios en ciencia, tecnología y sociedad (Jasanoff, 2004; Jasanoff, Kim, 2015). De manera específica, el artículo recurre a la noción de “promesas tecnocientíficas” (Joly, 2010), definidas como soluciones colectivamente construidas por distintos grupos de actores que incorporan la definición de problemáticas futuras, adversas y potencialmente irreversibles (Joly 2010). El concepto permite articular las necesidades de la emergencia sanitaria, las aspiraciones nacionales en tanto país semi periférico y la constitución material de estas expectativas, a través de coaliciones de actores que hacen que estas promesas se sostengan (Desrosièrres, 1990; Latour, 2017) y se realicen a través de capacidades tecnocientíficas.

Argumentamos que en Argentina se construyeron tres grandes promesas respecto a las vacunas covid-19: (a) la “promesa de acceso” a las vacunas, centrada en su compra como tecnología cerrada; (b) la “promesa tecnoproductiva”, anclada en la expectativa de autonomía industrial; y (c) la “promesa de ciencia soberana”, anclada en la histórica promesa de contribución del sistema científico local con la sociedad a través de conocimientos y tecnologías “útiles” (Kreimer, 2021; Gazquez, Bortz, Santos, 2022). Cada promesa y su artefacto vacunal emblemático fueron sustentados por diferentes coaliciones de actores y elementos materiales y simbólicos que movilizaron diferentes visiones de futuro, representaciones y agendas colectivas de lo que es bueno y deseable a través de la ciencia y tecnología (CyT), dando lugar a diferentes formas de gobernanza y aspiraciones sobre el rol del Estado y de la ciencia pública.

Este trabajo contribuye con la – aún poco estudiada – literatura sobre promesas tecnocientíficas en contextos periféricos (Kreimer, 2021, 2023). Se explora, en un escenario de emergencia, la constitución mutua entre la construcción colectiva de expectativas, así como el desarrollo de tecnologías, capacidades sanitarias y elementos ideológicos que forman parte de una idiosincrasia nacional. Estos moldean imaginarios colectivos de caminos y futuros deseados sobre la ciencia y la salud, materializados en dichas tecnologías (Jasanoff, Kim, 2015).

A continuación se presenta la metodología utilizada y el marco conceptual abordado y discutido. Posteriormente, se analizan tres promesas tecnocientíficas, cada una centrada en diferentes aspectos de las vacunas contra la covid-19 junto con las coaliciones de actores y elementos materiales asociados a cada una. Por último, se examinan las implicancias de estas promesas en los procesos de toma de decisiones y en el diseño de políticas públicas.

## Abordaje teórico-metodológico

Los desarrollos tecnológicos son concebidos o “imaginados” en función de las expectativas de futuros deseables, y los futuros adversos que ayudan a evitar (Joly, 2010; Adler, 1987). En este sentido, las tecnologías y expectativas de futuro están simbólica y materialmente interconectadas: es en las “imágenes de futuro” que se entrelazan los aspectos técnicos y sociales. Estas expectativas no son abstractas, sino que son materializadas por distintos actores clave en artefactos y sistemas tecnológicos (Borup et al., 2006). Circulan en discursos que movilizan elementos simbólicos, moldean lo que los distintos actores hacen y piensan, demarcan problemáticas y asignan sentidos vinculados a la CyT (Keller, 2011). Dentro de estos elementos simbólicos se incluyen los imaginarios, entendidos como formas socialmente compartidas de significar el mundo, que se materializan en acciones, representaciones, discursos, imágenes, artefactos, instituciones, leyes y valores (Castoriadis, 2003; Comastri, 2015). La CyT, en tanto conocimiento e institución, es movilizada ante situaciones de peligro e incertidumbre mediante nuevas intervenciones, basadas en ciertos imaginarios contextualmente arraigados que ofrecen un marco de interpretación de esa nueva realidad. Así, la CyT no solo funciona en términos de representación del orden natural, sino también que se origina y a su vez interviene en los órdenes social y cultural. En términos de Latour (2013), la relación entre lo material y lo imaginario no es una cosa o la otra, sino que están mutuamente interconectados y moldeados.

Las promesas tecnocientíficas, que incorporan expectativas e imaginarios situados, pueden entenderse como el marco que construye la futura forma y orientación de una tecnología en una comunidad sociopolítica, a través de soluciones compartidas e imaginadas colectivamente que buscan dar respuesta a problemáticas futuras y potencialmente adversas. Así, las promesas son un “imperativo de convergencia” para un propósito imaginado, construido colectivamente por coaliciones de actores alineados en pos de cierta problematización a la que se busca dar respuesta mediante la CyT.

Éstas se componen de una dimensión discursiva, generando horizontes de expectativas que buscan interesar a distintos actores atrás de un objetivo colectivo a gran escala. Se componen de elementos de legitimidad, es decir, la justificación de criterios para la asignación de recursos varios tras cierta solución pertinente y no de otra; y de credibilidad, siendo validadas por las coaliciones de actores sociales heterogéneos que sostienen la promesa (cuanto más grande la promesa, más atención atrae) (Joly, 2010). Las promesas generan una “pre-disciplina” de los imaginarios sociales y formas de concebir los desarrollos científico-tecnológicos en cierto ámbito social y de lo que es deseable emprender, de ahí su carácter performativo. Pero también son materializadas en la construcción de desarrollos científico-tecnológicos, impulsados por – y a la vez refuerzan – nuevas coaliciones entre distintos actores e intereses, instituciones, conocimientos, artefactos y otros elementos que contribuyen a la realización de las promesas y al “funcionamiento” de los artefactos que las materializan.

E ste t rabajo rec u r re a u na metodolog ía c ua l itat iva basada en ent rev istas semiestructuradas y en profundidad y en el análisis documental. Se realizaron 31 entrevistas a actores e informantes clave que participaron de distintas instancias de la compra, manufactura y/o desarrollo de vacunas covid-19 desde el sector industrial (diez entrevistas), científico (12 entrevistas, incluyendo asesores, inmunólogos, desarrolladores de vacunas y especialistas en ensayos clínicos), funcionarios públicos del sector sanitario y regulatorio (cuatro entrevistas), del sector científico-tecnológico y de innovación (CTI) e industrial (cuatro entrevistas) y de organismo sanitario internacional (una entrevista). Las mismas fueron transcriptas y codificadas para recuperar los tópicos más salientes. Por cuestiones éticas, no se mencionaron los nombres de los entrevistados. La reconstrucción de las coaliciones se realizó por método de bola de nieve, bajo la premisa de seguir a los actores (Latour, 2007).

El análisis documental tomó como componente central la cobertura periodística sobre las vacunas covid-19 en Argentina. Para esto se realizó una búsqueda sistemática de notas periodísticas en tres periódicos *online* de alta circulación (Boczkowski, Mitchelstein, 13 jun. 2023), dando cuenta de diversos posicionamientos políticos en el país. Se realizaron búsquedas sistemáticas de los artículos que hicieran referencia a las palabras clave “vacunas covid-19”, entre marzo del 2020 y octubre del 2023. Se recabó un corpus de 150 notas periodísticas, organizando en una matriz los discursos y representaciones referidos a las distintas vacunas, y a los roles del Estado, del sector científico público y del sector industrial.

La información recabada permitió periodizar la trayectoria de la construcción de expectativas y la respuesta del Estado argentino para la provisión de las vacunas de covid-19 en tres dinámicas superpuestas pero diferenciadas. La identificación de cada una y su dinámica está dada por un cambio en los elementos simbólicos movilizados (las expectativas, representaciones, factores de legitimidad y credibilidad), así como en los elementos materiales, cambiando el artefacto “vacuna covid-19” movilizado como emblemático (Joerges, 1999) y, con ello, la coalición de elementos que lo impulsan y lo hacen “funcionar” de manera situada (Bijker, 1997).

## La promesa de acceso a las vacunas

El acceso a las vacunas covid-19, fundamental para el éxito de la campaña de vacunación en un momento de alta demanda global, se convirtió en un objetivo primordial para el Ministerio de Salud (Minsal). Tras el inicio de los ensayos clínicos de los primeros candidatos vacunales en el segundo semestre de 2020, el Minsal inició negociaciones tempranas y simultáneas con varios proveedores de vacunas. A pesar de que Argentina se convirtió en un sitio preferencial para los ensayos clínicos de la vacuna Pfizer-BioNTech (Los detalles…, 7 ago. 2022) un conjunto de obstáculos retrasó la entrega de estas vacunas en el país. Estos incluyeron desde desacuerdos sobre los términos de adquisición, una solicitud de prórroga de jurisdicción y un compromiso de bienes soberanos por parte de la empresa multinacional (Thomas et al., 2021; Blanco, 15 dic. 2020), sistemas de precios escalonados para la pre-compra de vacunas, así como patrones globales de distribución asimétrica de las vacunas hacia las “periferias” y estrategias de “nacionalismos vacunales” (Blinder, Zubeldía, Surtayeva, 2021; Zhou, 2022).

Ante esta circunstancia, el Estado argentino avanzó en el proceso de adquisición de vacunas con un enfoque pragmático, bajo la premisa de lograr el acceso a la mayor cantidad de dosis posible en un contexto de alta demanda mundial. Esto derivó en acuerdos con siete empresas o instituciones internacionales, que permitieron planificar la estrategia en el Plan Estratégico de Vacunación contra la covid-19 en Argentina (Argentina, 2020). En el marco de estas demoras, en noviembre de 2020 el gobierno inició negociaciones con el Fondo Ruso de Inversión Directa para adquirir 25 millones de dosis de vacunas Sputnik V. Las primeras tres mil dosis arribaron al país el 24 de diciembre del 2020 (Pizzi, 15 jun. 2021), con la aerolínea de bandera del país, Aerolíneas Argentinas, transportando el primer lote de vacunas Sputnik V, como muchos otros en los meses siguientes, y dando al operativo un sentido de orgullo nacional:

Contar con una aerolínea de bandera le permite a nuestro país tener una respuesta rápida ante cada necesidad logística para que más ciudadanos estén protegidos y vacunados ([presidente de Aerolíneas Argentinas], Piloteado…, 18 abr. 2021).

Si no hubiéramos tenido una aerolínea de bandera, no sé si hubiéramos conseguido traer en esos primeros meses de pandemia lo que trajimos (entrevista 16, realizada el 16 ago. 2023).

En diciembre de 2020, la vacuna Sputnik V se convirtió en el primer artefacto emblemático de la promesa de acceso a las vacunas, posibilitando el inicio de la campaña de vacunación el 29 de diciembre de 2020 (Alberto…, 5 nov. 2020).

**Figura 1:**
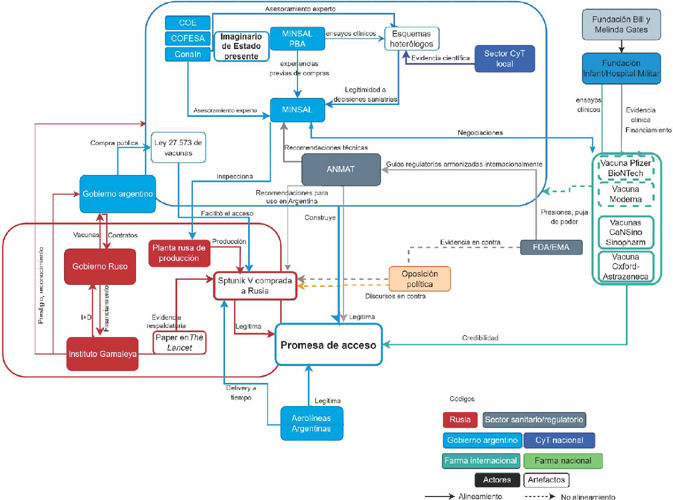
Coalición que sustenta la promesa de acceso a las vacunas (Fuente: elaborada por las autoras)

Los discursos de la urgencia en el acceso a vacunas y el rol rector del Estado en la gestión de la adquisición contribuyeron en la legitimación de esta promesa. Se buscó sustentar su credibilidad en el prestigio del Instituto Gamaleya en Rusia (Sputnik V…, 23 dic. 2020) y su experiencia previa en el uso de la tecnología adenoviral para diseñar vacunas, con una patente internacional por su trabajo en vacunas contra el Ébola. También se apeló a la autoridad y prestigio de la agencia regulatoria argentina Administración Nacional de Medicamentos, Alimentos y Tecnología Médica (Anmat), de referencia internacional, la cual evaluó la documentación enviada desde Rusia y avaló la eficacia y seguridad de la Sputnik (Kollmann, 20 ene. 2021). La Figura 1 muestra la coalición de actores y elementos materiales que dieron sustento y viabilidad a la promesa de acceso.

No obstante, distintos episodios relacionados a la adquisición de las vacunas contribuyeron a restar credibilidad al primer artefacto que encarnó la promesa de acceso: primero, la falta de aprobación de la Sputnik V por parte de la Organización Mundial de la Salud (OMS) y la Food and Drugs Administration estadounidense, agencia de referencia líder a nivel mundial. Segundo, cierta percepción de inexperiencia, al ser desarrollada por un agente externo al core mundial de empresas y países reconocidos por liderar la I+D en vacunas. Estos procedimientos despertaron suspicacias públicas, reflejadas en y alimentadas por la cobertura mediática (Alberto…, 5 nov. 2020; Demonte et al., 2024) en un contexto de polarización política y crisis económica (Abers, Rossi, Von Bülow, 2021). Así, se generó una percepción de tensión entre la noción de seguridad/riesgo sanitario y la noción de urgencia en el acceso. La publicación del artículo científico en *The Lancet*, en febrero del 2021, ejerció de efecto demostración que permitió apaciguar las controversias públicas al respecto (Logunov et al., 2021). Asimismo, la filtración de la aplicación de vacunas por fuera del Plan de Vacunación a personas con influencia política (conocido en Argentina como “vacunación VIP”) sumó un manto de opacidad al proceso de vacunación, signado por la grieta política interna (Demonte et al., 2024). En respuesta a estos cuestionamientos, el 24 de marzo del 2021 se lanzó el Registro Federal de Vacunación (Nomivac), plataforma de datos abiertos que buscaba transparentar y facilitar el acceso a información pública relativa al Plan Estratégico Nacional para la Vacunación.

Si bien en Argentina la vacuna contra la covid-19 como artefacto preventivo en sí no suscitó cuestionamientos en la arena público-mediática, dada la extendida percepción positiva de las vacunas en el país (Fundación Bunge y Born, 2021), la elección de qué artefactos vacunales específicos iban a ser aplicados sí fue motivo de debate público (Demonte et al., 2024; Zunino, Kessler, Vommaro, 2022; Cravacuore, 2021), así como atribuciones de responsabilidad en la esfera pública respecto a las decisiones gubernamentales durante la pandemia, en un contexto de grieta política preexistente.^3^

Al acuerdo con Gamaleya le siguieron nuevos acuerdos para garantizar la vacunación: con Astrazeneca, con el Serum Institute of India (vacuna ChAdOx1 nCoV-19, con tecnología transferida por Oxford/AstraZeneca), con el mecanismo Covax (vacuna ChAdOx1 nCoV-19 Oxford/AstraZeneca), con China (Sinopharm y CanSino) al inicio del 2021; y con empresas estadounidenses (Moderna y Pfizer) a mediados/fines del 2021. La urgencia de la adquisición de vacunas estaba asociada a la geopolítica de su distribución y a la limitada capacidad industrial, tanto de las empresas multinacionales como de empresas en India y China con limitaciones en la entrega por la falta de suministros en la cadena global de valor. Así, para abril de 2021, mientras que el gobierno argentino había comprado por adelantado 56 millones de dosis de diversas marcas, solo 5 millones habían llegado al país (Altindis, 2022).

La Figura 1 muestra la coalición de elementos movilizados en la construcción y materialización de la promesa de acceso. Se destaca el rol clave ejercido desde el sector sanitario como brazo ejecutor del gobierno nacional, dialogando con las autoridades sanitarias de los gobiernos provinciales, incluso de signos políticos opositores, y movilizando consejos de expertos científicos para la toma de decisiones en contextos de incertidumbre para la adquisición y administración de las vacunas en el territorio nacional. Además, distintos actores destacaron el excepcional trabajo mancomunado de funcionarios del oficialismo y de la oposición durante el 2020.

Sustentar esta promesa requirió construir y alinear diversas capacidades estatales que contribuyeron a la credibilidad de la promesa. La sanción de la ley 27.573, que declaró de interés público la investigación, desarrollo, fabricación y adquisición de vacunas covid-19, daba un marco legal a la adquisición y distribución de vacunas fuera de los esquemas regulares de compras del Minsal (por ejemplo, autorizando pagos adelantados, en divisa extranjera y por fuera de esquemas de licitación, los términos contractuales, y las aprobaciones regulatorias de emergencia) (Cravacuore, 2021). A esto se sumaron las distintas capacidades (políticas, regulatorias, logísticas) para el avance de la campaña de vacunación (Naciones Unidas Argentina, 2021), otro elemento legitimador de la promesa.

En esta promesa se vislumbra un sentido de soberanía que refiere a asegurar el acceso y derecho a la salud en tiempos de crisis, con el Estado como actor clave de estas acciones. A su vez, esta promesa y sus elementos legitimadores pusieron de relieve la necesidad de abordar la problemática respecto al acceso oportuno a vacunas, contribuyendo así a la construcción de legitimidad de las siguientes promesas.

## La promesa tecnoproductiva

Argentina cuenta con cuatro décadas de desarrollo de industria biotecnológica, generando capacidades que fueron abonando la competitividad del sector (Gutman, Lavarello, 2014; Stubrin, 2019). En la actualidad, cuenta con 340 empresas biotecnológicas, 102 de ellas en salud humana, ocupando el primer lugar en Latinoamérica y, según los últimos datos publicados en base a la metodología de la Organización para la Cooperación y el Desarrollo Económicos, el décimo lugar a nivel mundial (Stubrin et al., 2023). En agosto de 2020, la empresa biofarmacéutica mAbxience, que integra el grupo empresario Insud, firmó un acuerdo de transferencia tecnológica para fabricar en Argentina 150 a 250 millones de dosis del principio activo de la vacuna Oxford/AstraZeneca (Quién es…, 12 ago. 2020; Astrazeneca…, 12 ago. 2020). El acuerdo, fruto de una negociación entre privados impulsada desde la Fundación Slim, estipulaba que el llenado y envasado (*fill and finish*) estaría a cargo del laboratorio mexicano Liomont. El acuerdo constituyó una apuesta empresarial, en la medida que tanto la transferencia como la producción local iniciaron antes de la aprobación de emergencia de la vacuna por parte de las agencias regulatorias de referencia a nivel mundial.

Paralelamente, la promesa de acceso a las vacunas a través de la Sputnik V se vio amenazada, tanto por la liberación gradual de los lotes como por la falta del segundo componente de la vacuna. En este marco, en enero del 2021, la empresa india Hetero Labs Limited puso en contacto al Fondo Ruso de Inversión Directa con el Laboratorio Richmond, con quienes tenía trayectoria de trabajo conjunto, para la producción local de la vacuna Sputnik V (Blanco, 26 feb. 2021). El acuerdo estipulaba que el laboratorio Richmond se encargaría de la formulación de la vacuna, con el principio activo importado desde Rusia. Para el envasado, Richmond sub-contrató a la empresa MR Pharma ubicada en el partido de Malvinas Argentinas (Buenos Aires), con cierta experiencia como empresa manufacturera de biológicos por contrato. Aunque la iniciativa también fue presentada como un acuerdo entre privados, Richmond hizo hincapié en el apoyo gubernamental recibido (Beato Vassolo, 8 jun. 2021).

La promesa se legitimó mediante discursos que enfatizaron: primero, la relevancia de lograr autosuficiencia en vacunas, considerando el contexto de Argentina como un país semi-periférico y reforzando, consecuentemente, la expectativa de un acceso más rápido y amplio en el futuro (El laboratorio…, 26 feb. 2021). La empresa mAbxience, al fabricar el principio activo de la vacuna de AstraZeneca, cubrió el 75% del suministro acordado para el país. Sin embargo, la distribución siguió los esquemas pactados por AstraZeneca con cada país, revelando las desventajas en gobernanza de la producción local de vacunas bajo licencias externas.

La credibilidad de la promesa, en un contexto en el cual solo un reducido número de países tenían capacidad de manufactura,^4^ se fundamentó en la “notable experiencia y trayectoria productiva” de las empresas, así como la posibilidad de “producir vacunas para abastecer a nivel regional y global” (El gobierno…, 4 jun. 2021). Estos argumentos permitieron a estas empresas alinear a diversos actores y ampliar sus capacidades de producción de biológicos (Las razones…, 17 feb. 2023; Pérez, 15 sep. 2023; Blanco, 13 jul. 2023).

Los discursos del sector (bio)farmacéutico local apuntaron a destacar su relevante contribución a la salud pública y su fructífera relación con el sector CyT público. En un escenario de histórica desconfianza en Argentina hacia el sector empresarial (López, 2006; Donato, 18 oct. 2018), la búsqueda de legitimación privada se alineó con los discursos que destacaron su disposición a innovar y realizar inversiones significativas para contribuir al sector sanitario nacional.

En tér minos mater ia les, los ac uerdos de t ra nsferenc ia tec nológ ica pa ra la manufactura local derivaron en nuevas capacidades en las empresas en el marco de la emergencia (Lundval, Johnson, 1994): tecnológicas (puesta a punto de nuevas líneas productivas en biológicos, ampliación de sus horizontes productivos), industriales (nuevas instalaciones y equipamiento), comerciales (nuevos mercados, visibilidad, posicionamiento como marca) y estratégico-políticas (vinculación con otros actores clave, llegada al gobierno para allanar procesos de importación de insumos). Algunas de éstas, a su vez, reforzaron capacidades estatales. Tal es el caso de Richmond, que transfirió a Anmat el *know-how* del análisis de los lotes de Sputnik V tras recibir los protocolos del Instituto Gamaleya.

En la Argentina, por ejemplo, todas las facilidades que nos dieron para importar equipamiento que nosotros necesitábamos que normalmente es bastante dolor de cabeza ellos lo allanaron [gobierno] (entrevista 8, realizada el 7 jul. 2023).

[La falta de vacunas Sputnik] puso en el escenario una foto real de la necesidad de tener producción local, de las dificultades que se generaban en todos los mercados por la falta de producciones locales. A raíz de la pandemia conocimos otro montón de actores que nos abren las puertas a nivel internacional, que nos acercó un montón de actores, y eso me empezó a generar otro tipo de vínculos que nos permitieron, me están permitiendo ahora, cerrar todos estos acuerdos [de transferencia tecnológica] (entrevista 10, realizada el 12 jul. 2023).

**Figura 2:**
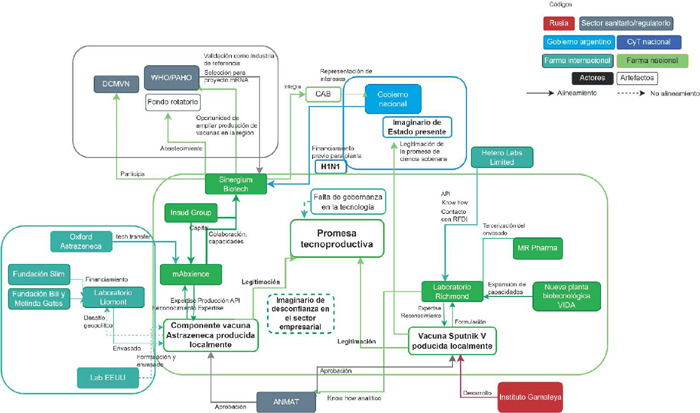
Coalición de elementos que sostiene la “promesa tecnoproductiva” (Fuente: elaborada por las autoras)

La Figura 2 muestra la coalición de actores y elementos materiales que dieron sustento y viabilidad a la promesa de producción local de vacunas. Esta figura visibiliza el rol clave ejercido por el sector (bio)farmacéutico privado nacional en la materialización de esta promesa, en diálogo con actores clave del ámbito internacional.

En abril del 2021, la OMS lanzó el Programa de Transferencia de Tecnología de ARNm, con centro en Sudáfrica, orientado a que beneficiarios de países de bajos y medianos ingresos recibieran capacitación y las licencias necesarias para acceder a la tecnología (Avanza…, 15 feb. 2023). La convocatoria obtuvo más de treinta expresiones de interés. En septiembre de 2021, la Organización Panamericana de la Salud (OPS) y la OMS seleccionaron a Bio-Manguinhos (Fundación Oswaldo Cruz) en Brasil y a la empresa Sinergium Biotech (Grupo Insud) en Argentina (Using…, 2021). Si bien esta última no contaba con capacidad para producir principios activos – a diferencia de mAbxience –, sí contaba con una década de experiencia en adopción de tecnologías, control de calidad y *fill and finish* de vacunas biotecnológicas para provisión al Estado nacional (entrevista 3, realizada el 24 mayo 2023).

La promesa tecnoproductiva profundizó la promesa de acceso a las vacunas frente a la asimetría global de su distribución, a través de la expectativa por la producción local como camino hacia la autosuficiencia sanitaria: “Desarrollar políticas públicas sustentables dirigidas a promover el desarrollo de vacunas y otros productos biotecnológicos, expandiendo nuestras capacidades de producción de estos insumos estratégicos y contribuyendo a la autosuficiencia sanitaria regional” (Argentina avanza…, 13 mar. 2023).

Si bien el sector privado fue el principal movilizador de elementos que dieron viabilidad a la promesa (Figura 2) (tecnologías, convenios de vinculación, cadenas de proveedores, financiamiento, relación con OPS/OMS), el Estado operó como portavoz y legitimador de la misma: desde lo discursivo, destacando las capacidades de la industria biofarmacéutica nacional como claves para garantizar el acceso a vacunas y medicamentos en el contexto de emergencia sanitaria, y posicionando las transferencias tecnológicas como hitos clave para la “autonomía sanitaria”.

La larga trayectoria y altos estándares de calidad de la industria farmacéutica argentina, que cuenta con 200 plantas de producción, de las cuales 160 son de capitales nacionales y 40 del sector público. Nuestra región cuenta con las capacidades esenciales necesarias para ampliar la producción regional y disminuir las brechas en el acceso a las vacunas ([ministra de Salud], Una empresa…, 21 sep. 2021).

Nosotros, cuando al principio hablábamos de soberanía, no hay más soberanía sanitaria que tener transferencia de tecnología, que tener ciencia para generar nuestras propias respuestas, para poder dar esas respuestas a la región (entrevista 16, realizada el 16 ago. 2023).

Desde lo material, se destacó el contexto científico-tecnológico favorable promovido por el Estado: institucionalidad, conocimientos, recursos humanos calificados, financiamiento a la asociatividad público-privada, que permitió acumular capacidades para sustentar la expectativa de autoabastecimiento. Desde lo regulatorio, la calificación de Anmat por OPS como agencia sanitaria de referencia para la región permitió tanto un tratamiento expedito de las vacunas importadas como la auditoría de las empresas locales bajo estándares de calidad que permitan comercializar y exportar vacunas. De las 131 millones de dosis recibidas por Argentina durante la pandemia, 30,2 millones fueron manufacturadas en Argentina (22,5 mAbxience, 7,7 Richmond).

No obstante, ciertos episodios contribuyeron a restar legitimidad a la promesa. Por un lado, ciertos miembros de la oposición política resaltaron la participación del sector privado en la producción local de vacunas como un negocio entre el gobierno y ciertos actores del sector privado (bio)farmacéutico, alimentando el escepticismo local en la relación gobierno/sector privado (Sesión completa…, 19 mayo 2021). Por otro lado, la falta de gobernanza sobre la tecnología y la cadena de provisión de insumos^5^ generaron tanto demoras en el aprovisionamiento nacional como sobre la decisión de distribución de los lotes manufacturados en el país, tras los acuerdos previos de la matriz. Esto puso en jaque la cultura política de “Estado presente” garante del acceso a la salud: “La necesidad de autosuficiencia de la región durante la pandemia nos hizo despertar a todos” ([ministra de Salud], Vizzotti…, 8 feb. 2023).

Así, esta promesa se movilizó para legitimar una promesa más grande de “ciencia soberana”, que llevó a construir y alinear diversos elementos clave, como se explicitará a continuación.

## La promesa de ciencia soberana

Tras declarar la emergencia sanitaria nacional, distintas instituciones de CyT nacionales – el Ministerio de Ciencia, Tecnología e Innovación Productiva (Mincyt), el Consejo Nacional de Investigaciones Científicas y Técnicas (Conicet), la Agencia Nacional de Promoción de la Investigación, el Desarrollo Tecnológico y la Innovación (Agencia I+D+i), el Minsal y el Ministerio de Desarrollo Productivo – conformaron la Unidad Coronavirus, un espacio de coordinación sin precedentes que buscó dar apoyo a proyectos CyT del sector público relacionados o que pudieran reorientarse al desarrollo de tecnologías para enfrentar la pandemia (Castiglione, Barberón, Bacchi, 2023). Los esfuerzos se centraron en promover y financiar proyectos CyT del sector público relacionados con diagnóstico, control, prevención, tratamiento y monitoreo. Inicialmente la posibilidad de desarrollar localmente vacunas fue desestimado, debido a la falta de antecedentes en el país en este tipo de desarrollos *ex novo* y la presunción de inviabilidad en el contexto de emergencia sanitaria: “Los evaluadores dijeron no, cómo vamos a apoyar proyectos de vacunas si no vamos a llegar a ningún lado, [hacer] una vacuna es muy difícil” (entrevista 17, realizada el 17 ago. 2023). Para diciembre de 2022, Argentina presentaba seis desarrollos endógenos de vacunas covid-19, que se sumaron a las capacidades de transferencia tecnológica y fabricación que resultaron críticas en 2021 (Bortz, Sanmartin, 2024). En este trabajo nos centraremos en el análisis del proyecto Arvac, cuyo candidato vacunal recibió la aprobación de Anmat.

En el marco de la convocatoria de financiamiento IP-Covid (IP Covid-19…, 2020), uno de los 125 proyectos financiados fue “Desarrollo de herramientas que contribuyan a la prevención de la infección por SARS-CoV 2”, liderado por la doctora Juliana Cassataro, investigadora principal del Conicet radicada en el Instituto de Investigaciones Biotecnológicas de la Universidad Nacional de San Martín (IIB-Unsam). El grupo tenía para entonces una extensa trayectoria en el desarrollo de adyuvantes innovadores para vacunas contra diversas enfermedades locales, incluyendo dengue y brucelosis. Para diciembre del 2020, el proyecto había logrado avanzar exitosamente en la formulación de un prototipo vacunal inyectable.

A partir de estos resultados promisorios, en abril de 2021, el Fondo Argentino Sectorial (Fonarsec), de la Agencia I+D+i), realizó una convocatoria para el fortalecimiento de capacidades en fase pre-clínica para ensayos *in vivo* de vacunas argentinas covid-19 (Nuevos…, 3 sep. 2021). Según fue declarado por entrevistados, esta convocatoria fue organizada para apoyar financieramente al proyecto de la doctora Cassataro, y a la vez diversificar la promoción a otros tres proyectos de vacunas desarrollados desde institutos del Conicet.

La convocatoria del Fonarsec^6^ para estudios *in vivo* asignó al grupo del IIB – así como a los otros proyectos – sesenta millones de pesos (aproximadamente, seiscientos mil dólares estadounidenses) para avanzar en el desarrollo de una vacuna basada en tecnología de proteínas recombinantes. Esta tecnología se había utilizado en las vacunas contra la hepatitis B y el VPH, ambas incluidas en el Calendario Nacional de Vacunación y fabricadas en el país. Ésta se destacaba por ser una tecnología de alta inmunogenicidad, baja incidencia de efectos secundarios y segura para niños y embarazadas (Pasquevich et al., 2023). El prototipo vacunal fue denominado “Arvac-Cecilia Grierson”, en honor a la primera doctora en medicina del país.

En noviembre del 2020, tras una serie de conversaciones con potenciales empresas adoptantes, la Agencia I+D+i contactó al Laboratorio Pablo Cassará, empresa (bio) farmacéutica de capital nacional con una extensa trayectoria en desarrollo industrial, validación y manufactura de vacunas y otros productos biológicos. La empresa poseía desarrollos previos en proteínas recombinantes para vacunas contra hepatitis B y antirrábica, y terapias de avanzada (entrevista 31, realizada el 13 dic. 2023). Desde diciembre del 2020, Unsam y Cassará trabajaron en un proceso conjunto que involucró la escalabilidad del desarrollo y la reformulación del adyuvante utilizado inicialmente en el prototipo vacunal.

Hacia fines de 2021, entre los desarrollos locales mencionados, la candidata Arvac fue la que tuvo mayor grado de avance. El prototipo vacunal fue significado por el equipo de trabajo como una vacuna de refuerzo (o de segunda generación) “segura y tradicional”. Al ser una tecnología madura, su selección apuntó a un tratamiento expeditivo y a la rápida aprobación por parte de la Anmat, además de su adaptabilidad a nuevas variantes del virus. El diseño apuntó además a cumplir con los criterios de vacunación pediátrica y para embarazadas (Blanco, 13 dic. 2021; Pasquevich et al., 2023), la existencia de capacidad de producción local a gran escala y su adecuación a las condiciones de distribución en el vasto territorio nacional al requerir condiciones de refrigeración estándar (2° a 8°C) (Blanco 30 mar. 2022).

La Fase 1 de ensayos clínicos de Arvac comenzó en abril 2022, financiada por 7,5 millones de dólares por el Laboratorio Cassará. Las Fases 2 y 3 comenzaron en enero del 2023, financiadas por el Fonarsec, de la Agencia I+D+i, con 8 millones de dólares (Comienza…, 6 mar. 2023) y dirigidas por la empresa de ensayos clínicos iTRIALS. La vacuna recibió la aprobación de Anmat el 17 de octubre del 2023 por disposición 2023-8604 (La primera…, 18 oct. 2023). A la fecha de la redacción de este trabajo, el primer lote estaba siendo inspeccionado por la autoridad sanitaria. Esto constituyó un hito nacional, no solo por el nivel de inversión estatal alcanzado para un proyecto, sino porque Arvac se convirtió en la primera vacuna preventiva para enfermedades infecciosas diseñada y desarrollada íntegramente en Argentina (Concluyo el…, 31 ago. 2022). Además, la convirtió en una de las vacunas menos costosas en su desarrollo a nivel mundial: 16 millones de dólares, mientras que en 2021 la empresa Pfizer declaró un costo de I+D de mil millones para su vacuna Conmirarty (Kaplan, Wehrwein, 2021).

El desarrollo de la vacuna Arvac suscitó gran expectativa desde el sistema científico y sanitario nacional. En un país en crónico estado de crisis y con un promedio de 65% de la I+D financiada con fondos públicos,^7^ Arvac contribuyó a materializar la promesa subyacente al contrato de la CyT argentina con su sociedad, es decir, de generar soluciones a problemas públicos relevantes para el país a través de tecnologías útiles, con un objeto políticamente resonante. Esta es una temática históricamente arraigada en la trayectoria del sistema CyT nacional (Sanmartin, 2023), movilizada en la actualidad como argumento de legitimación de la relevancia del sector.

El desarrollo Arvac fue celebrado en redes sociales por distintos integrantes del sistema CyT, así como declarado de interés nacional por las Cámaras de Diputados y de Senadores de la nación. La inédita coordinación de esfuerzos estatales desde los sistemas CyT y sanitario, que viabilizaron el desarrollo, movilizaron discursivamente el imaginario del “Estado presente” y de la capacidad del estado de movilizarse ágilmente:

Se confirma el círculo virtuoso entre la investigación, el desarrollo y el impacto positivo en la sociedad ... la investigación con desarrollo y el impacto positivo en la salud de los argentinos ([ministra de Salud], Esteban, 31 mar. 2021).

Es un orgullo para la ciencia argentina disponer de una vacuna nacional hecha por nuestras científicas y científicos, y lo que significa llevar la investigación y el desarrollo al servicio de las personas ([ministra de Salud], Esteban, 31 mar. 2021).

El estado presente y la decisión política de un gobierno para articular con todos los ministerios ([ministra de Salud], La primera…, 18 oct. 2023).

Un segundo núcleo discursivo de legitimidad, que contribuyó a justificar la movilización de recursos y capacidades, fue la expectativa de construir capacidades para la futura sustitución de importaciones de otras vacunas pasibles de ser desarrolladas a partir de esta plataforma. Las vacunas implican un déficit comercial significativo (más del doble de importaciones que exportaciones del sector) (Plan Argentina Productiva 2030, Ministerio de Economía). Esto permitía además cuantificar los posibles retornos de la inversión pública en ciencia, en beneficio de la sociedad: “Es un día histórico de la ciencia argentina porque permitirá sustituir la importación de vacunas y exportarlas. Argentina importa vacunas por 500 millones de dólares al año y este desarrollo va a significar un gran paso para sustituir importaciones” (Aprobaron…, 18 oct. 2023).

La promesa de ciencia soberana, centrada en Arvac como artefacto emblemático y representativo de la política de promoción a la CyT, ganó credibilidad movilizando discursivamente la “excelencia y prestigio” de los grupos de investigación argentinos y las capacidades biotecnológicas disponibles en el país, aun cuando en periodos iniciales algunos científicos y *policy makers* consideraran la inviabilidad de un proyecto de este tipo en el país:

Demostramos que tenemos científicos con capacidad de producir desarrollos de alta calidad ([Guillermo Docena, desarrollador de Argenvac], Luna, 7 ago. 2021).

La pandemia ha demostrado las capacidades latentes que teníamos y la potencia que tiene la articulación de políticas públicas en esos ámbitos ([presidente de la Agencia I+D+i], Villarroel, 13 nov. 2021).

Estas capacidades se vieron centradas en el equipo de 12 personas lideradas por Cassataro, pero también con el involucramiento de más de seiscientos investigadores y profesionales y las capacidades (científicas, industriales, políticas, clínicas, organizativas) de 24 instituciones públicas y privadas de todo el país (Flynn, 15 nov. 2023). Asimismo, la actividad asociativa con el Laboratorio Pablo Cassará, involucrando el co-desarrollo y licencia de la tecnología para producción, se constituyó en un caso de éxito resonante de las políticas CTI.

**Figura 3:**
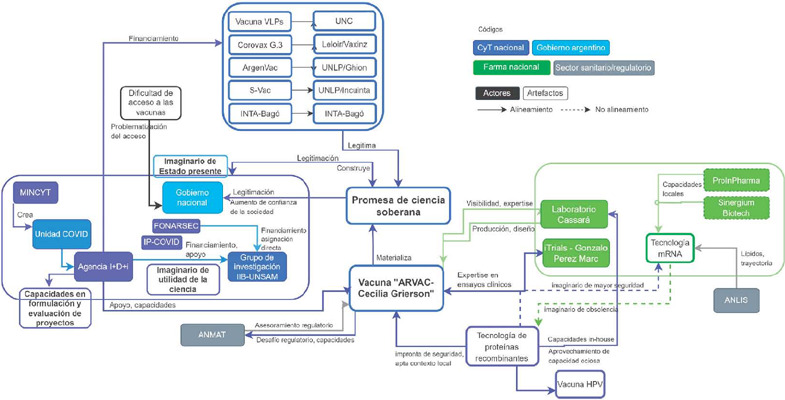
Coalición de elementos que conforman la “promesa de ciencia soberana” (Fuente: elaborada por las autoras)

La Figura 3 muestra la coalición de actores y elementos materiales que dieron sustento y viabilidad a la promesa de ciencia soberana, impulsada predominantemente desde el sector CTI. Los distintos actores clave de la coalición alinearon y construyeron un conjunto de capacidades locales (tecnológicas, regulatorias, de vinculación), en busca de experiencias futuras de desarrollo de vacunas.

Cada paso que se dio hizo un camino para nuevas investigaciones ([ministra de Salud], Blanco, 18 oct. 2023).

Para el país, encabezar un procedimiento como la elaboración de una tecnología sanitaria tan sensible como una vacuna, implica la puesta en marcha de capacidades muy valiosas. Habilidades, infraestructura y recursos ([ministra de Salud], Esteban, 5 ago. 2022).

[El proyecto Arvac] deja una plataforma que va a posibilitar transferir la tecnología para el desarrollo de otras vacunas ([ministro de CTI], La primera…, 18 oct. 2023).

Por un lado, diversas capacidades construidas desde el sector público viabilizaron el proyecto. En la Agencia I+D+i esto incluyó desde la agilización de la evaluación de proyectos (implementando la evaluación de ideas, contrapuesta a la evaluación tradicional de proyectos), hasta la la vinculación del grupo del IIB con Cassará, teniendo en cuenta las capacidades de producción del socio privado y su experiencia en vacunas. Mientras que la Unsam consolidó un equipo administrativo para la ejecución ágil del financiamiento, la Anmat realizó un acompañamiento excepcional al proyecto en cuanto al diseño de los ensayos clínicos y allanar el camino de su aprobación.

La disponibilidad de investigación clínica de calidad internacional en el país, con estructura y capacidad de reclutamiento de voluntarios, permitió que la vacuna fuera aprobada por la autoridad regulatoria en tiempo récord. La promesa de alcanzar un hito histórico posibilitó que iTRIALS y los sitios clínicos aceptaran un costo inferior a los percibidos en ensayos habituales, posibilitando la realización de las fases 2 y 3 con el financiamiento disponible.

Por otro lado, la participación en el proyecto Arvac también le permitió al Laboratorio Cassará adquirir *expertise* tecnológica (escalamiento de procesos) y regulatoria en materia de vacunas, sumando estos aprendizajes a los adquiridos en su trayectoria previa. El laboratorio recibió premios y reconocimiento por su labor en el proyecto, lo que le permitió adquirir visibilidad y establecer vinculaciones clave con distintos actores (entrevista 31, realizada el 13 dic. 2023).

Esta promesa fue el marco y condición de posibilidad de la movilización de diversos elementos clave (actores, capacidades de distinto tipo, intereses, imaginarios, expectativas, recursos etc.) que abren el camino a concretar promesas inconclusas del pasado respecto al desarrollo y producción de vacunas en el país: “Tenemos que pararnos desde esta plataforma [Arvac] para pensar nuevos problemas y desafíos como por ejemplo el dengue o la fiebre amarilla” (entrevista 21, realizada el 20 nov. 2023). Pero también supuso un hito en la trayectoria CyT nacional, al materializar la convergencia de las expectativas de distintos sectores traducidas en un objetivo común.

## Discusión

La pandemia de covid-19 impulsó un realineamiento entre los actores del sector CyT, del gobierno y de la industria (bio)farmacéutica, convirtiéndose en un marco fructífero para los proyectos científicos nacionales orientados a misiones (Mazzucato, 2018). Los desarrollos CyT (vacunas pero también *kits* de diagnóstico, mascarillas, análisis epidemiológicos etc.) se convirtieron en portadores de visiones de gobernanza sobre lo que el Estado argentino debía ser y hacer con el objetivo de reducir la incertidumbre en condiciones de emergencia sanitaria.

El análisis permitió identificar tres formas superpuestas de coproducción entre conocimiento y formas de gobernanza. Cada una se centró en distintas promesas tecnocientíficas, materializadas en distintos artefactos emblemáticos “vacuna covid-19”, que marcaron niveles progresivos de autonomía en términos de incorporación local de conocimiento, desde la adquisición de vacunas como paquete cerrado, la manufactura local, mediante transferencia tecnológica, hasta la capacidad de internalización completa del ciclo. Cada una fue sustentada por coaliciones diferentes y movilizando actores, capacidades, inscripciones materiales, discursos e imaginarios que las sostuvieron como promesas (con mayor o menor grado de realización). Estos elementos fueron performativos al dejar antecedentes y aprendizajes para desafíos futuros.

Por un lado, resulta indiscutible el eje rector del Estado como impulsor de las dinámicas de la primera y tercera promesas. Mientras que la expectativa central en la primera era el acceso a la salud, aún en condiciones de fuerte dependencia internacional, en la tercera se apeló a la gobernanza de las tecnologías sanitarias para garantizar el acceso. Las tres promesas tecnocientíficas sostienen y abrevan del imaginario de “Estado presente”, esgrimido desde el gobierno (opuesto a un “Estado neoliberal ausente”).^8^ El Estado contribuyó, de forma más explícita o implícita, a la construcción de las distintas promesas, movilizando discursiva y materialmente elementos (capacidades, experticias, financiamiento, vinculaciones etc.) en la legitimación de sus decisiones en materia de salud. Esto se condice con el imaginario del bienestar y el acceso a la salud asegurado por el Estado, que se inscribe como un derecho constitucional (art. 42 de la Constitución Nacional), y se materializa a través del financiamiento y la promoción de la salud y la CTI (con niveles dispares de éxito) (Bortz, Sanmartin, 2024; Spinelli, 2010).

Según se destacó en varias de las entrevistas, los conocimientos y experticias específicas de distintos funcionarios – enmarcados en un gobierno autodefinido públicamente como “de científicos, no de CEOs” (Néspolo, 1 mar. 2020) – resultaron clave para traccionar a otros actores y elementos clave para el funcionamiento de las promesas. Es a través de las acciones de materialización de las promesas que se institucionalizan estas prácticas y experticias. Es decir, se vuelven regulares y continuas en el tiempo generando irreversibilidad (Levy, 1996). Además, los altos funcionarios de las instituciones no solo le proporcionan el “saber hacer” y el “saber qué”, sino también el “saber dónde”, es decir, las creencias, expectativas y objetivos que muestran el camino, una manera particular de identificar los problemas y sus soluciones. Esto resulta clave en el marco de las promesas, las cuales embeben el “saber dónde” y permiten la movilización de elementos clave para su concreción. También permiten hacer esas prácticas y experticias durables en el tiempo, al establecer antecedentes y aprendizajes.

Respecto al sector CyT público, la comunidad científica movilizó tanto conocimiento experto como desarrollos tecnológicos clave para sobrellevar la pandemia. Así, intentó reivindicar su relevancia y utilidad social, al tiempo que legitimó la capacidad del propio Estado para enfrentar un escenario de crisis superpuestas (sanitaria, socioeconómica y política). Imaginarios de ciertas formas de gobernanza de la ciencia (“ciencia como política de Estado”, “ciencia nacional al servicio de la nación”), de histórica trayectoria en el país (Comastri, 2015), se coprodujeron con las múltiples tecnologías aportadas desde este sector durante la pandemia. Particularmente en el diseño de Arvac, artefacto emblemático de la promesa de ciencia soberana, se inscribieron dimensiones del contexto local, materializando así una promesa “periférica”: la utilización de una tecnología “oportuna”, localmente “adecuada” (en términos de capacidades locales de producción, facilidad de logística y evidencia de seguridad) en contraposición a las tecnologías basadas en ácidos nucleicos (mRNA y vectores adenovirales) preferidas por las grandes multinacionales:

Es algo intermedio, es un enfoque, si se quiere, de tecnología conveniente, la que nos conviene a nosotros para resolver un problema. Es adecuada a nuestro contexto, pertinente, diríamos, desde un enfoque sociotécnico, porque nosotros tenemos un talón de alquiles, tenemos un eslabón débil que es la logística. Tenemos que pararnos desde esta plataforma para pensar nuevos problemas y desafíos (entrevista 21, realizada el 20 nov. 2023).

Existen vacunas basadas en tecnología más innovadoras. Nosotros buscamos ser efectivos antes que originales. El enfoque fue de ‘tecnología conveniente’ ([presidente de la Agencia I+D+i], Peirano, 21 oct. 2023; destacados en el original).

La coordinación entre Estado y sector privado implicó una convergencia de expectativas: el horizonte de soberanía sanitaria movilizado desde el Estado, embebido en esta promesa y convertido en compromiso de acción, requería de la coordinación del sector privado y sus capacidades en un rol explícito y activo para la materialización de la promesa en una tecnología vacunal funcional al contexto local.

Por otro lado, el “éxito” de la promesa tecnoproductiva sirvió como elemento legitimador del lugar clave otorgado por el Estado al sector privado de cara a la coalición sustentadora de la promesa de ciencia soberana. En este sentido, en esta tercera dinámica, el Estado buscó interesar y alinear a un sector privado nacional que, como se ha reportado en la literatura, suele poseer poco interés en la inversión para el desarrollo y producción local de vacunas por el alto riesgo financiero y la complejidad del proceso, en comparación a otros productos biológicos terapéuticos (Jensen, Barry, Kelly, 2023).

## Consideraciones finales

Este trabajo nos permitió explorar las coaliciones de elementos heterogéneos que sustentan a las promesas asociadas a las vacunas covid-19 (su adquisición, fabricación, desarrollo), pero también profundizar en los repertorios de legitimación para la toma de decisiones y los patrones cambiantes de gobernanza. Las distintas promesas dan cuenta de una coproducción entre tecnologías y visiones de futuros deseables, a través de artefactos emblemáticos y de las políticas significadas y materializadas en estos artefactos inherentemente políticos. Se buscó con esto contribuir a la escasa literatura sobre promesas tecnocientíficas en países periféricos (Kreimer, 2021, 2023). En particular, se muestra la condición de posibilidad para el cumplimiento de dichas promesas, materializándose en una tecnología (en este caso, la vacuna Arvac) que moviliza discursos e imaginarios en torno al “estado presente” y al rol clave de la ciencia para la sociedad.

El anhelo argentino de “soberanía” fue construido y movilizado históricamente de distintas formas por distintos grupos de actores como elemento legitimador, desde científicos, la sociedad, hasta gobernantes, en tanto representantes de la voluntad soberana de los ciudadanos (Comastri, 2020; Hagood, 2006; Vilas, 1997; Grimson, 2019). Este repertorio discursivo recupera la concepción del Estado como comunidad política imaginada y de acción colectiva, que revaloriza lo “nacional”, en contraposición a los discursos de apertura pro-mercado (Jessop, 2010). La construcción del imaginario de soberanía, en tanto reconocimiento de un “nosotros” en construcción y redefinición de roles estatales, interpela discursivamente a diversos espacios de la vida social presente, incluyendo el CyT (La Serna, 2013), en la búsqueda de producción de nuevos significados de identidad.

Así, cada promesa mostró una materialización específica y progresiva de “soberanía”. Primero, a través de la compra de un bien social inestable, que dependía de proveedores externos y arcas crónicamente vacías. Segundo, a través de una industria local parcialmente autónoma, en medio de una desconfianza subyacente entre los sectores público y privado y una integración subordinada en las cadenas de valor de las vacunas. Tercero, a través de una vía última de autonomía tecnocientífica, que restablece la centralidad y prestigio de una ciencia nacional, en asociación con la industria farmacéutica como actores de poder, como vía hacia la soberanía sanitaria con dominio tecnológico pleno. En este sentido, el sector CyT público busca constituirse identitariamente como valor social clave, a la vez que circulan nuevos sentidos asociados a las dinámicas CyT/Estado/sector privado. Entre ellos se destacan la participación, la flexibilidad, la interdisciplinariedad, la conexión del dominio social con el tecnológico, el elogio de la investigación y la formación continua, la colaboración entre universidad y empresas, la horizontalización de las jerarquías, el trabajo en equipo y la organización en red (Vera, 2017).

Este trabajo buscó brindar herramientas para repensar la relación entre el Estado y la CyT (en tanto conocimiento y como institución), recuperando la dimensión epistémica y material en los procesos de toma de decisiones y el diseño e implementación de políticas públicas con miras a sistemas sanitarios más resilientes, contribuyendo a la literatura latinoamericana (Gadelha, 2003; Gadelha et al., 2020; Natera, Tommasini, Vera Cruz, 2019; Monteiro, Roth, Shelley-Egan, 2023; Fonseca, Shadlen, Achcar, 2023). Se destaca que con la ciencia local de prestigio y calidad, y a pesar de los vaivenes político-económicos, se lograron formular y movilizar estrategias – interesando y alineando a actores clave *a priori* desconectados entre sí – tras un fin sanitario concreto. El análisis respecto a las formas en que los distintos actores significaron y resignificaron los fines y los medios en un contexto de urgencia y de cara a la construcción de realidades pos pandémicas, permite echar luz sobre las dinámicas de coproducción de los órdenes social y material y el rol clave de la CTI en estos procesos.

## NOTAS

1 Antecedente del Instituto Nacional de Microbiología (1957), luego Administración Nacional de Laboratorios e Institutos de Salud “Dr. Carlos G. Malbrán” (1996).

2 Para 2010, se estimaba que 79% de las unidades de vacunas importadas y el 96% de los dólares gastados fueron en empresas multinacionales. Este perfil contrasta con el escenario de Brasil, en donde la provisión de vacunas se realiza en un 94% a través de las capacidades de manufactura de laboratorios públicos, en particular Fiocruz y el Instituto Butantan (Gadelha et al., 2020).

3 Si bien esta temática es un objeto de reflexión en sí, la misma no es abordada en el presente trabajo, sino que problematizamos la significación y movilización de las vacunas, en tanto artefactos políticos, por parte de grupos expertos y desde el gobierno nacional.

4 China, Estados Unidos, Alemania, India, Reino Unido, Países Bajos, Rusia, Suiza, Corea del Sur, Brasil, Sudáfrica, Argentina (Using…, 2021).

5 A escala mundial, los principales países exportadores de insumos manufactureros para vacunas son Alemania, Estados Unidos y China (Using…, 2021).

6 El Fonarsec, uno de los instrumentos de financiamiento público de la Agencia I+D+i, busca acelerar el desarrollo de proyectos público-privados, así como crear o expandir centros de investigación orientados al sector productivo en áreas consideradas estratégicas: salud, energía, agroindustria, desarrollo social, ambiente y cambio climático a través de tecnologías transversales y convergentes (TICs, nanotecnología, biotecnología).

7 Según datos del Sistema Integrado de Indicadores del Mincyt (Indicadores de I+D…, 2023).

8 Particularmente, la movilización del concepto de “Estado presente” se agudizó en contraposición al “retiro” del Estado durante las políticas pro-mercado de los años 1990 y la movilización de referencias identitarias y sentidos, como el individualismo, la lógica empresarial en la esfera política, la inversión privada como fuente de transparencia en la gestión pública y la planificación estratégica como medio de conducir las sociedades al desarrollo (Vera, 2017).
